# Operational Considerations in Global Health Modeling

**DOI:** 10.3390/pathogens10101348

**Published:** 2021-10-19

**Authors:** Katherine M. Broadway, Kierstyn T. Schwartz-Watjen, Anna L. Swiatecka, Steven J. Hadeed, Akeisha N. Owens, Sweta R. Batni, Aiguo Wu

**Affiliations:** 1 Defense Sciences, Inc. (DSI), Support to DTRA Technical Reachback, San Antonio, TX 78230, USA; katherine.m.broadway.ctr@mail.mil; 2 Applied Research Associates (ARA), Support to DTRA Technical Reachback, Albuquerque, NM 87110, USA; kierstyn.t.schwartz-watjen.ctr@mail.mil; 3 Battelle Memorial Institute, Support to DTRA Technical Reachback, Columbus, OH 43201, USA; anna.l.swiatecka.ctr@mail.mil (A.L.S.); steven.j.hadeed.ctr@mail.mil (S.J.H.); 4 Defense Threat Reduction Agency (DTRA), Fort Belvoir, VA 22060, USA; akeisha.n.owens.civ@mail.mil (A.N.O.); sweta.r.batni.civ@mail.mil (S.R.B.)

**Keywords:** biosurveillance, DTRA, epidemiological modeling and simulation, global health operations

## Abstract

Epidemiological modeling and simulation can contribute cooperatively across multifaceted areas of biosurveillance systems. These efforts can be used to support real-time decision-making during public health emergencies and response operations. Robust epidemiological modeling and simulation tools are crucial to informing risk assessment, risk management, and other biosurveillance processes. The Defense Threat Reduction Agency (DTRA) has sponsored the development of numerous modeling and decision support tools to address questions of operational relevance in response to emerging epidemics and pandemics. These tools were used during the ongoing COVID-19 pandemic and the Ebola outbreaks in West Africa and the Democratic Republic of the Congo. This perspective discusses examples of the considerations DTRA has made when employing epidemiological modeling to inform on public health crises and highlights some of the key lessons learned. Future considerations for researchers developing epidemiological modeling tools to support biosurveillance and public health operations are recommended.

## 1. Introduction

Biosurveillance, defined as the collection, analysis, and communication of information pertaining to biological threats, fits synergistically with epidemiological modeling and simulation to support real-time decision-making during public health emergencies. It serves as a valuable asset for a prompt response to emerging infectious diseases, such as Ebola and COVID-19. As almost 75% of emerging diseases are zoonotic [1], animal health surveillance is an important component of a robust biosurveillance system. Animal and human data collected from biosurveillance systems can be leveraged to model the geographical spread of disease over time and help answer a wide variety of operational questions, such as trends of disease spread, estimates of morbidity and mortality rates in affected populations, and impact of pharmaceutical and non-pharmaceutical interventions on disease transmission. The analysis and interpretation of data generated by epidemiological models can further improve biosurveillance systems. 

For public health operational purposes, biosurveillance systems are crucial to risk assessment and subsequent risk management. The Department of Defense (DoD) and associated agencies play an important role in this process. Specifically, the Armed Forces Health Surveillance Branch (AFHSB) was established to meet the needs of the DoD with functional areas in surveillance, reporting, technology evaluation, quality improvement, coordination of biosurveillance efforts, and epidemiological investigations [2]. Under AFHSB, the Global Emerging Infections Surveillance (GEIS) section was established for improving the surveillance of diseases as well as enhancing the prevention of and response to infectious diseases [3]. Another DoD agency, the Defense Threat Reduction Agency (DTRA) has sponsored the development of numerous modeling and decision support tools as well as analytic capabilities to address questions of operational relevance in response to emerging epidemics and pandemics. As a national defense agency, the major function of DTRA is to invest in technical innovations to counter weapons of mass destruction (WMD) and asymmetrical threats, while responding to urgent needs that shape United States (U.S.) capabilities [4]. Emerging infectious diseases have posed immediate threats to public health and military operations. As a result, DTRA has been involved in a variety of public health responses, regularly answering requests for information (RFIs) from authorized, official government partners using unique technical expertise and modeling capabilities. The Chemical and Biological Technologies Department at DTRA developed the Biosurveillance Ecosystem (BSVE) to provide technology and software informatics solutions to advance the science and address critical gaps in real-time biosurveillance, early warning, and decision support for biological threats [5]. BSVE development, led by a team of software architects at Digital Infuzion (https://www.digitalinfuzion.com/ accessed on 13 October 2021) [6], began in 2012, two years prior to the 2014 West African Ebola epidemics. The unclassified BSVE system automatically scans open-source biological threat-related data streams, integrates these disparate data sets into a common repository, and provides a virtual analyst workbench for end-users to access different DTRA-funded software applications and user interfaces for customized data analytics and visualizations. Analytic tools hosted by the BSVE include capabilities for disease risk mapping, agricultural and animal population data analytics, outbreak prediction, early warning, and forecasting disease spread capabilities during a biological event. Additionally, the analyst virtual workbench facilitates active data collaboration among users through dossiers, joint case reporting, and chat functions. 

The BSVE is a cloud-based capability hosted by Amazon Web Services. By design, the features of the BSVE enable automation of information and user display customization for end-users, regardless of their geographic location or skill level. A Software Developers Kit (SDK) provided guidelines to support the backend software architecture and processing pipelines, thus ensuring different application developers built data and analytics in a format compatible for ease of transfer to the BSVE. Industry, academia, and the national lab partners developed functional applications for biological threat agent modeling capabilities, which were hosted in the BSVE for all registered end users. Active research and development continued through 2019 at which point the BSVE was transitioned from DTRA to the Department of Homeland Security (DHS) for sustainment and maturation. 

Outlined below are a few examples of the contributions and considerations DTRA made when employing epidemiological modeling to inform public health crises. 

## 2. Western Africa and the Democratic Republic of the Congo (DRC) Ebola Outbreaks

DTRA provided extensive support during the Ebola outbreaks in West Africa (2014–2016) and the DRC (2018–2020). The RFIs encompassed a variety of efforts across the DoD including the Combatant Commands (COCOMS), the National Guard Bureau (NGB) as well as other government agencies. The weekly technical information briefs capitalized on input from various government and private organization sources including DTRA’s BSVE capability. These briefs contributed to biosurveillance communication and covered available science about the virus, disease transmission, significant local events that affected response efforts, available diagnostics, medical countermeasures, vaccines, PPE, and decontamination practices. DTRA actively participated in interagency modeling development through participation in working groups with the Biomedical Advanced Research and Development Authority (BARDA) and the Centers for Disease Control and Prevention (CDC). In partnership with the Virginia Polytechnic Institute and State University, DTRA employed the Comprehensive National Incident Modeling System program (CNIMS) [7]. CNIMS utilizes an agent-based model and realistic network simulations to predict possible disease spread that could affect U.S. military and civilian populations, both domestic and abroad. These agent-based models incorporate geographically identified synthetic populations, where an “agent” complete with demographic attributes, behaviors and activities, a household, and other social interactions represents each person. The synthetic populations were developed using census data, time-use surveys, building-use data, and transportation networks. CNIMS employed compartmental models to simulate disease transmission through the synthetic population. Unique compartments in the modeling system were essential to understanding alternative routes of transmission identified during the West Africa outbreak, namely nosocomial transmission in healthcare settings and transmission during certain funeral practices. These models highlighted the essential need for contact tracing and preventing these important modes of transmission to be able to successfully mitigate the Ebola outbreaks.

## 3. COVID-19 Pandemic

Since the beginning of the unprecedented COVID-19 pandemic, multiple modeling teams from all around the world have been requested to provide epidemiological models to support preparedness and containment efforts. As one of those teams, DTRA has received a large number of questions ranging from technical information to modeling projections, with many requests having come early in the pandemic. The DTRA modeling team has projected the spread of COVID-19 infections in 83 countries and answered over 600 RFIs. The requests originated from various agencies and organizations across the U.S. federal government, including the White House, DoD, DHS, Department of Health and Human Services (HHS), U.S. military branches, and COCOMS. Some RFIs were also released to support operations with government agencies from allied countries. DTRA provided daily technical briefs and risk assessments, which helped to guide interpretation, and monitoring for new global biosurveillance data. Frequently updating the modeling parameters ensured the results were representative of the current situation including, control measures and scientific advancements and discoveries. Regular monitoring of country-specific data on medical capacity and incorporation of the data into the models improved the quality of the results. The model results anticipated future outbreak locations across the world, especially vulnerable areas at greatest risk, allowing stakeholders to make informed operational decisions regarding personnel and resources.

Two modeling tools sponsored by DTRA, EpiGrid [8] and PatchSim [9], were used extensively to answer modeling questions regarding disease transmission during the COVID-19 pandemic. EpiGrid, originally developed as part of the BSVE by Los Alamos National Laboratory, is an epidemiological modeling tool. The Joint Science and Technology Office (JSTO) of the Chemical and Biological Defense Program (CBDP) at DTRA funded its development. EpiGrid is a geographically resolved, extended compartmental model, capable of simulating the temporal and spatial spread of epidemics in near real-time [8,10]. This capability was used to answer time-sensitive requests, such as quick turn-around RFIs. PatchSim, sponsored by DTRA and developed by the Virginia Polytechnic Institute and State University, was employed for less time-sensitive requests that required high-resolution data, such as county-level modeling. PatchSim is a well-utilized, extended compartmental model which calibrates multiple county-level scenarios into an ensemble and produces adaptive forecasts using machine learning [9,11,12]. 

In addition, EpiGrid’s two-population modeling capability compares disease transmission dynamics between subsets of individuals, such as civilians vs. military, by integrating interaction rates between the two populations. This capability proved to be very useful during the operational planning of military missions. This feature can be applied to any distinct population of interest, including groups residing in congregate residential settings, such as nursing homes, college campuses, or prisons. In order to better represent the full impact of the COVID-19 pandemic in various countries, DTRA used these two tools to further develop epidemiological models that take into account concurrent crises, such as military conflicts, earthquakes, hurricanes, floods, and epidemics of different infectious diseases.

## 4. Utility of DTRA Modeling and Operational Support

DTRA is uniquely positioned to advise various decision-makers in the U.S. and partner nations during states of emergency and disasters, including endemic, epidemic, pandemic, and emerging disease spread events. DTRA’s analytical and modeling capabilities are applied across tactical and strategic perspectives, depending on the event-phase and actionable response under consideration. DTRA’s scientific forecasting efforts provide requestors with necessary analyses to maintain situational awareness, and in specific circumstances, provide actionable information. The temporal and geographic granularity of DTRA’s modeling tools is crucial features that inform operations. DTRA is one of many sources used for infectious disease modeling in the DoD, with others including AFHSB, Joint Program Manager-Transformational Medical Technologies (JPM-TMT), Naval Research Laboratory (NRL), National Center for Medical Intelligence (NCMI), Naval Health Research Center (NHRC), Office of the Assistant Secretary of Defense (Health Affairs) (OASD(HA)), and academic partners [13]. The products provided by DTRA span the entirety of disease response efforts. At critical stages, the products have been used to inform decision-makers within the DoD and across the federal government on key operational determinations. Past examples of DTRA’s contributions include, but are not limited to, the following:

- Analyses generated for the DoD during the initial response phase of the Ebola outbreak aided in site selection for new treatment centers. These products were updated continuously to maintain situational awareness.
- Analyses provided at the beginning of the COVID-19 pandemic helped senior DoD personnel assess potential impacts of disease spread on medical logistics and support efforts by civilian authorities.
- Disease forecasts and county-level estimates of infection provided to National Guard units aided their evaluation and anticipation of resource requirements and deployments.
- Modeling parameters, data, and investigative reports freely shared with other US agency modeling groups, specifically HHS and CDC, helped develop a consensus and best practices for modeling efforts.

## 5. Lessons Learned and Improvements for the Future

Past disease outbreaks and the ongoing COVID-19 pandemic show that anticipation and preparedness are critical steps of effective public health response, as opposed to a reactive approach. DTRA continues to build new capabilities for overcoming future threats based on previous experiences and lessons learned.

*Managing insufficient data collection and reporting*: Access to high-quality, trustworthy data sources is integral in forming the foundation for biosurveillance systems and accurate emerging infectious disease modeling. However, data reported by various health systems can be of low fidelity. Heterogeneity in data continues to be a major source of information bias and a challenge for epidemiological modeling of infectious diseases across various settings and populations. Poor quality data can be the result of many factors such as underestimating cases due to insufficient data collection, test sensitivity, and specificity, tracking the prevalence of the outbreak, public health infrastructure challenges, and unfamiliarity with the reporting system. Additionally, delays and sensitivities in establishing a uniform case definition and diagnostic criteria make it difficult to compare data across healthcare settings, especially on an international level. 

To account for these challenges in reporting and to approximate the real situation, several workaround strategies must be considered. First, developing a uniform method for approximating the number of infected individuals from reported cases and deaths will help overcome issues of under- and overestimation of infections across specific regions and models. Second, using both official and open media reports to estimate the arrival time of the first case in the modeled country should also be considered. This ensures that simulation start dates account for transmission that occurs before the first reported case, improving the temporal resolution of the epidemiological model. Third, scaling parameters from previously calibrated epidemiological models to new countries using global scoring systems like the Infectious Disease Vulnerability Index [14], provides consistency between country-specific models. Simple scaling metrics also increase time efficiency. The global scoring systems and the scaled parameters should be representative of several country-specific measures that include but are not limited to, healthcare infrastructure and public health capacity metrics. The true extent of the COVID-19 pandemic disease spread is unknown, which creates challenges for the validation of modeling strategies. Mitigating insufficient data collection and reporting in near real-time also presents challenges. In an effort to ensure the quality of simulations and analysis results, DTRA calibrated each specific country or regional model to reported deaths and approximated infections over the entire duration of the pandemic. The epidemiological models, used during the COVID-19 pandemic, were projected forward 6-weeks or less due to the potential inability to capture future changes in viral transmission, policy, and social behaviors. Finally, various sources of information on sociodemographics and government response efforts were incorporated in the models to direct disease transmission dynamics. These sources provided information on complex trends and the effects of public health measures across a range of platforms that included Google Community Mobility data, Apple Mobility Trends report, and public surveys.

*Quick response to requirements*: A short turn-around time is essential for real-world event requests, which poses a challenge to several modeling paradigms. DTRA maintains a careful balance between a timely response, data resolution, and quality. Elements of DTRA are uniquely positioned to meet time-sensitive deadlines due to the support provided 24 hours per day, 7 days per week to warfighters, government officials, and first responders [7,13]. The DTRA capabilities are further improved by the investments made in biosurveillance and modeling research and development.

*Communication of modeling results*: Importantly, modeling products must be accessible to a wide variety of audiences from subject matter experts and key decision-makers to people without epidemiological or public health expertise. From an operational and modeling design point of view, clearly understanding the purpose of the product and the questions that need to be answered are critical steps of model development. Similarly, presenting data clearly and concisely is imperative for the efficient dissemination of information. In that vein, DTRA developed an online, interactive dashboard for authorized users throughout the DoD and the U.S. government to access COVID-19 modeling results. At one point the dashboard had more than 6100 visitors [15] and was frequently updated with new data to ensure modeling products represented the real-world situation. Additionally, users were able to interact with the data to create analyses and visualizations that fit their individual needs. DTRA continues to advance technologies and devote expertise to improving the functionality of its analyses.

## 6. Conclusions

Sensible investment and improvement to a global biosurveillance system will enable a rapid and innovative response to the next pandemic or epidemic. Multidisciplinary expertise in the fields of epidemiology, microbiology, clinical medicine, animal health, social sciences, and modeling and simulation is required for the construction of a robust, comprehensive biosurveillance system. Early actions using the biosurveillance system can provide timely information and decision support for policymakers and operational decision-makers, potentially preventing an outbreak from escalating to a pandemic. Increased environmental and diagnostic testing, expansion of current zoonotic surveillance systems, and building public health capacity in developing nations will support the functionality of biosurveillance systems globally. A well-designed biosurveillance system can enhance collaboration between international government partners and allied nations, especially for uniting outbreak responses and increasing global security. DTRA is uniquely positioned to invest in the long-term development of such capabilities and is equipped to adapt and transition those capabilities towards the next emerging infectious disease response. 

## Data Availability

Not applicable.

## References

[1] Gebreyes W.A., Dupouy-Camet J., Newport M.J., Oliveira C.J., Schlesinger L.S., Saif Y.M., Kariuki S., Saif L.J., Saville W., Wittum T. (2014). The global one health paradigm: Challenges and opportunities for tackling infectious diseases at the human, animal, and environment interface in low-resource settings. PLoS Negl. Trop. Dis..

[2] Integrated Biosurveillance. https://health.mil/Military-Health-Topics/Combat-Support/Armed-Forces-Health-Surveillance-Branch/Integrated-Biosurveillance.

[3] Global Emerging Infectious Surveillance. https://health.mil/Military-Health-Topics/Combat-Support/Armed-Forces-Health-Surveillance-Branch/Global-Emerging-Infections-Surveillance-and-Response.

[4] Defense Threat Reduction Agency Directorates. https://www.dtra.mil/MissionDirectorates/.

[5] Stark K.A., Shah A., Borgman J., Somborac M., Carson J., Hauser L., Kola K., Virkar H. (2019). Data Science, Analytics and Collaboration for a Biosurveillance Ecosystem. Online J. Public Health Inform..

[6] DTRA Scientists Develop Cloud-Based Biosurveillance Ecosystem. https://www.defense.gov/News/News-Stories/Article/Article/681832/dtra-scientists-develop-cloud-based-biosurveillance-ecosystem/.

[7] Dembek Z.F., Mothershead J.L., Chekol T., Myers D.B., Meris R.G., Meranus D., Wu A. (2017). Operational Perspective of Lessons Learned from the Ebola Crisis. Mil. Med..

[8] Fenimore P., McMahon B., Hengartner N., Germann T., Mourant J. (2018). A Suite of Mechanistic Epidemiological Decision Support Tools. Online J. Public Health Inform..

[9] Venkatramanan S., Bhattacharya P., Porebski P., Klahn B. (2020). PatchSim. https://github.com/NSSAC/PatchSim.

[10] Mourant J.R., Fenimore P.W., Manore C.A., McMahon B.H. (2018). Decision Support for Mitigation of Livestock Disease: Rinderpest as a Case Study. Front. Vet. Sci..

[11] Venkatramanan S., Sadilek A., Fadikar A., Barrett C.L., Biggerstaff M., Chen J., Dotiwalla X., Eastham P., Gipson B., Higdon D. (2021). Forecasting influenza activity using machine-learned mobility map. Nat. Commun..

[12] Venkatramanan S., Chen J., Fadikar A., Gupta S., Higdon D., Lewis B., Marathe M., Mortveit H., Vullikanti A. (2019). Optimizing spatial allocation of seasonal influenza vaccine under temporal constraints. PLoS Comput. Biol..

[13] Chretien J.P., Gaydos J.C., George D., Sanchez J.L., McCollum J.T., Pavlin J.A., Russell K.L. (2014). Epidemiologic modeling in the Department of Defense: Capability and coordination opportunities. Mil. Med..

[14] Moore M., Gelfeld B., Okunogbe A., Paul C. (2017). Identifying Future Disease Hot Spots: Infectious Disease Vulnerability Index. Rand Health Q..

[15] DTRA Supports Global Health Efforts through Modeling and Simulation. https://www.dvidshub.net/news/388606/dtra-supports-global-health-efforts-through-modeling-and-simulation.

